# Interactions between zoonotic pathogens and infectious disease spread: Why understanding mechanisms and modelling matters more than ever

**DOI:** 10.1016/j.bsheal.2025.07.008

**Published:** 2025-08-07

**Authors:** Naizhe Li, Sunxiao Ruan, Huaiyu Tian

**Affiliations:** a School of National Safety and Emergency Management, Joint International Research Laboratory of Catastrophe Simulation and Systemic Risk Governance, National Key Laboratory of Intelligent Tracking and Forecasting for Infectious Diseases, Beijing Normal University, Beijing 100875, China; b State Key Laboratory of Remote Sensing and Digital Earth, Beijing Key Laboratory of Surveillance, Early Warning and Pathogen Research on Emerging Infectious Diseases, Beijing Research Center for Respiratory Infectious Diseases, Center for Global Change and Public Health, Beijing Normal University, Beijing 100875, China

**Keywords:** Pathogen interactions, Mathematical modelling, Zoonotic diseases, Cocirculation dynamics, Public health strategies

## Abstract

Interactions among zoonotic pathogens play a critical role in shaping disease transmission, severity, and public health responses. However, the mechanisms and population-level consequences of these interactions remain underexplored in current modelling frameworks. This review aims to synthesize emerging evidence and address key scientific challenges in understanding how pathogen interactions influence transmission dynamics and mathematical modelling, with a focus on zoonotic and other cocirculating pathogens. In this review, we synthesize current evidence on synergistic, antagonistic, and neutral interactions between zoonotic and other cocirculating pathogens. We explore the underlying mechanisms of these interactions, such as transmission enhancement, immune modulation, and resource competition, at both the individual and population levels. We further review mathematical models to illustrate how these interaction features, such as transmission pathways, coinfection histories, cross-immunity, and superspreading potential, could be incorporated into epidemiological frameworks to increase our understanding of the community transmission of infections. Particular attention is given to the challenges of parameter estimation, incomplete surveillance data, and the difficulty of modelling interactions across scales and pathogen types. Understanding and modelling these interactions is essential for predicting outbreak trajectories, designing effective vaccination strategies, and improving early-warning systems. We conclude by calling for enhanced integration of empirical data and mechanistic modelling, especially in the context of emerging zoonoses and postpandemic preparedness. This review provides a structured perspective to support future interdisciplinary efforts aimed at managing cocirculating pathogens and mitigating their public health impact.

## Highlights

• Three types of interactions—synergistic, antagonistic, and neutral—shape disease severity, transmission, and public health responses.
• Mechanisms such as transmission enhancement, immune modulation, and resource competition drive pathogen coexistence and competition.
• Mathematical models simulate multipathogen dynamics and address challenges in parameter estimation and data gaps.
• We call for enhanced surveillance, system-level modelling, and adaptive strategies to better manage cocirculating pathogens.

## 1 Introduction

Zoonotic infectious diseases are significant contributors to the global disease burden, and the pathogens causing these diseases possess considerable zoonotic potential because of their ability to infect various animal hosts, from which they can subsequently be transmitted to humans. Over recent decades, numerous epidemics caused by zoonotic pathogens have occurred, including those associated with severe acute respiratory syndrome coronavirus (SARS-CoV), A/H1N1 influenza viruses, Middle East respiratory syndrome coronavirus (MERS-CoV), dengue virus (DENV), and Nipah virus, among others. As of December 2024, the ongoing coronavirus disease 2019 (COVID-19) pandemic had resulted in at least 777 million cases and 7 million deaths worldwide [1,2]. The continuous circulation of emerging and reemerging zoonotic infectious diseases highlights the challenges in their prevention and control. Consequently, the cocirculation of pathogens—whether zoonotic or not—may become increasingly frequent, complicating transmission dynamics at both the individual host and population levels [[3], [4], [5]].

Understanding interactions between pathogens is crucial for elucidating the complex dynamics of infectious disease transmission and the factors driving epidemics. In this context, “interaction” refers to any process in which infection caused by one pathogen alters the likelihood, timing, or progression of infection caused by another pathogen. These interactions can significantly influence the spread, severity, and outcomes of diseases, thereby shaping public health responses and disease control strategies [[6], [7], [8]]. Research on interactions between different pathogens or between distinct strains of the same pathogen is vital for developing more effective approaches to control infectious diseases, especially in an era when emerging multipathogen threats are becoming increasingly challenging in terms of global health [9,10].

Mathematical models are employed to investigate the underlying mechanisms and effects of interactions between cocirculating pathogens at the population level to understand the cocirculation of pathogens. Such models provide a framework for integrating observations from surveillance data and extrapolating these insights to broader patterns beyond the immediate observations. In this review, we introduce the currently available evidence on the interactions of cocirculating pathogens, including those between zoonotic pathogens and between zoonotic and nonzoonotic pathogens, and the proposed mechanisms underlying these interactions. Both individual- and population-level evidence is included, as individual-level interactions serve as the biological foundation for understanding and modelling transmission dynamics at the population level. Although vector-borne diseases differ from diseases caused by most zoonotic pathogens, they are also included in our discussion. Additionally, we summarize key mathematical modelling approaches used to study pathogen interactions at the population scale, highlighting existing challenges and suggesting directions for future research.

## 2 Evidence of interaction

Increasing evidence has revealed interactions between various zoonotic pathogens and non-zoonotic pathogens. For any given pair of pathogens, their interaction can be classified into three main types, namely, synergistic, antagonistic, and neutral interactions. In a synergistic interaction, two pathogens enhance each other's effects, as observed for human immunodeficiency virus (HIV), which weakens the immune system of the host and significantly increases the risk of *Mycobacterium tuberculosis* infection [8]. In an antagonistic interaction, one pathogen suppresses the proliferation of another through immune responses or resource competition. For example, influenza virus infection may inhibit bacterial proliferation by altering the immune response of the host [11]. Neutral interactions occur when there is no significant effect between two pathogens, and their infection processes proceed independently. In this review, we detail both established and potential evidence for these interactions.

### 2.1 Synergistic interactions

Synergistic interactions between pathogens occur when coinfection leads to more severe disease than would be expected from individual infections. In these interactions, two pathogens enhance each other's ability to infect the host or exacerbate the severity of the disease. One possible explanation for such interactions is that the initial infection can alter the host environment, creating conditions that promote colonization by other bacteria or viruses. For example, influenza viruses may increase the permeability of mucosal barriers or trigger inflammatory responses that facilitate the entry of other pathogens [12]. Furthermore, influenza infection can impair the host immune response, increasing the vulnerability of an individual to subsequent infections [13].

A well-established example of a synergistic interaction is the interaction between the influenza virus and *Streptococcus pneumoniae*. Influenza infection can increase bacterial adherence to respiratory epithelial cells and disrupt host defence, thereby promoting the transition from asymptomatic carriage to severe pneumococcal disease [14,15]. This interaction is considered a major contributor to the increased incidence of secondary bacterial infections during influenza outbreaks [16]. Studies have also demonstrated that influenza infection impairs the clearance of methicillin-resistant *Staphylococcus aureus* (MRSA) in animal models, thereby increasing the severity of MRSA infections [17]. Influenza infection weakens the immune response, reducing the ability of the body to combat MRSA, which facilitates MRSA persistence and growth. Similarly, synergistic interactions have also been observed between influenza viruses and *Haemophilus influenzae* [18], *Neisseria meningitidis* [19], and *Mycobacterium tuberculosis* [20].

The coinfection of humans with avian and seasonal influenza virus subtypes provides potential evidence for synergistic interactions [[21], [22], [23]]. Previous studies have shown that avian influenza viruses predominantly replicate in the lower respiratory tract (LRT) in humans, whereas seasonal influenza viruses tend to infect the upper respiratory tract [[24], [25], [26]]. This coinfection suggests that the upper respiratory tract may serve as a potential site for reassortment, although further laboratory evidence is still needed.

In the case of coinfection with severe acute respiratory syndrome coronavirus 2 (SARS-CoV-2) and influenza A virus (IAV), experimental studies in animal models have indicated that combined infection leads to increased disease severity [27]. This includes increased weight loss and, in some cases, higher mortality rates than those caused by monoinfection with either virus alone [28]. Studies involving ferrets, hamsters, and mice showed that animals with SARS-CoV-2 and IAV coinfection experienced more severe disease than those infected with either pathogen alone, as evidenced by greater weight loss [[29], [30], [31], [32], [33], [34], [35], [36], [37]]. In some studies, this coinfection also resulted in higher mortality rates [29,31,34].

Synergistic interactions have also been observed for vector-borne diseases [38,39]. For example, DENV and Zika virus (ZIKV), which share a common vector, *Aedes aegypti*, exhibit geographical and seasonal cocirculation [40,41]. A critical mechanism in DENV–ZIKV coinfections is antibody-dependent enhancement (ADE), in which antibodies against one virus facilitate infection by the other virus. Studies have shown that anti-DENV antibodies can promote ZIKV infection [[42], [43], [44]] and vice versa [45,46], which may not only increase disease severity but also contribute to the geographical expansion and persistence of these viruses.

### 2.2 Antagonistic interactions

Antagonistic interactions occur when coinfection with two pathogens leads to lower than expected disease severity. These interactions arise when one pathogen inhibits the infection or replication of another pathogen, which occurs through various mechanisms, such as competition for host resources, immune system interference, or direct pathogen-pathogen interactions. For example, influenza viruses, similar to many other respiratory viruses, often trigger a nonspecific immune response (e.g., cytokine production and activation of innate immune cells), which can limit the ability of other pathogens to infect or proliferate [47,48]. Moreover, the immune response induced by an initial infection may restrict the ability of subsequent pathogens to cause infection.

The interactions between influenza viruses and respiratory syncytial virus (RSV) are frequently cited as an example of antagonism, with some studies suggesting that influenza viruses can delay or inhibit RSV infection. During the second wave of the 2009 influenza pandemic in France, the RSV epidemic was delayed, potentially due to immune responses triggered by the influenza virus [49,50]. However, this antagonistic interaction has not been universally observed, as other studies have shown that coinfection with influenza virus and RSV can result in more severe outcomes [51]. Similarly, during the 2009 pandemic, a rhinovirus epidemic in France coincided with a delay in the second wave of influenza infection, suggesting that rhinovirus might have exerted an antagonistic effect on influenza transmission [52]. However, such delays were not observed in other countries, indicating that interactions between these viruses may be affected by local factors, such as variations in reporting or public health measures. Furthermore, studies have shown that parainfluenza infections are less common in the presence of influenza infections, suggesting a competitive interaction between these viruses [53]. Nonetheless, the severity of coinfection can vary, and not all studies find that influenza and parainfluenza infections interfere with one another [54,55].

While the majority of evidence points to more severe disease during coinfection with IAV and SARS-CoV-2, antagonistic interactions have also been reported in several studies. For example, a history of infection with IAV—particularly when IAV precedes SARS-CoV-2 infection—has been shown to reduce the SARS-CoV-2 viral load in the LRT, suggesting an antagonistic relationship in which IAV impedes SARS-CoV-2 replication [28]. Similarly, a study revealed that vaccination with a live-attenuated influenza vaccine before SARS-CoV-2 infection reduced the SARS-CoV-2 viral load in ferrets [56].

Epidemiological studies have also provided evidence of antagonistic interactions, in which a history of respiratory infections appears to moderate the severity of SARS-CoV-2 infection. A retrospective cohort study indicated that a history of infection with endemic human coronaviruses (hCoVs) was associated with protection against COVID-19-related intensive care unit (ICU) admission [57]. This suggests that exposure to certain respiratory pathogens could induce immune system adaptations, such as trained immunity, which may offer cross-protection against other viral infections. Additionally, chronic infection with *Mycobacterium tuberculosis* has been linked to lower SARS-CoV-2 viral loads, indicating a potential antagonistic effect in which the presence of tuberculosis may limit SARS-CoV-2 replication and reduce disease severity in mice [58].

### 2.3 Neutral interactions

Neutral interactions occur when coinfection with two pathogens does not affect the severity or progression of the disease compared with monoinfection. Some studies have identified neutral interactions between SARS-CoV-2 and other respiratory pathogens, in which case the presence of one virus does not significantly alter the disease course or viral load of the other. For example, the prevalence of coinfection with SARS-CoV-2 and other pathogens (such as influenza viruses or human coronaviruses) has yielded mixed results in human population studies [59]. Some research has suggested that coinfection does not significantly affect SARS-CoV-2 detection [60] or hospitalizations [61], implying that these pathogens may coexist without affecting each other's replication or disease outcomes.

Additionally, studies that investigated the roles of previous respiratory infections, such as upper respiratory infections (URIs), in the susceptibility to SARS-CoV-2 reported conflicting findings. One study reported that prior URIs were associated with a lower risk of SARS-CoV-2 infection [62], whereas another reported an increased risk of SARS-CoV-2 infection [63]. These inconsistencies could indicate neutral interactions, in which case the pathogens coexist independently, with little to no significant effect on each other, depending on factors such as immune status or pathogen virulence.

However, these results must be interpreted with caution. First, the variability across studies may be due to factors such as the infection order, infection duration, and differences in animal models, highlighting the complexity of interactions. Second, confounding variables, such as comorbidities, could bias the findings, and many studies were not specifically designed to infer interactions [28]. Further research is needed to clarify the nature of these interactions and their implications for public health.

## 3 Mechanisms of interactions between pathogens

We next focus on three distinct hypotheses that describe the underlying mechanisms of pairwise interactions between pathogens, namely, transmission enhancement, immune modulation, and resource competition. These hypotheses help elucidate the diverse ways in which cocirculation can affect the dynamics of infectious diseases and have been widely considered in population-level models.

### 3.1 Transmission enhancement due to host and environmental factors

The transmission of one pathogen can be increased if the host has been recently infected with another pathogen. This phenomenon represents a critical mechanism of synergistic pathogen interactions, in which primary infection modifies the internal environment of the host in ways that promote colonization, replication, or shedding of the secondary pathogen [[64], [65], [66]].

The extent of transmission enhancement often depends on clinical symptoms and disease severity. For example, pathogens transmitted via respiratory routes may benefit from inflammation-induced coughing or sneezing during coinfection [67,68]. Clinical severity can also increase opportunities for pathogen dispersal. HIV infection depletes T cells, weakening the host’s immune response and leading to more severe and prolonged secondary infections, thereby increasing the likelihood of disease transmission [69,70]. Furthermore, the severity of disease can affect host behaviour (e.g., increased immobility or healthcare contact), which in turn affects transmission dynamics. For example, individuals with severe influenza symptoms often stay home, reducing their usual contact behaviour and lowering their risk of acquiring or transmitting additional infections [71,72]. In contrast, those with milder symptoms may continue normal activities, potentially facilitating both onward transmission and coinfection [73,74].

Disease progression models can be developed to predict the severity and progression of individual cases [75]. These models can identify patients who are more likely to require admission to the ICU or experience complications, thereby helping optimize treatment strategies. Machine learning models have been used to predict the likelihood of hospitalization in cases of coinfection with influenza viruses and SARS-CoV-2 [76]. However, current models do not adequately predict whether critically ill patients also experience coinfection during pandemics caused by viral infections, which remains a significant gap in the prediction of severe outcomes during viral outbreaks.

Environmental factors play significant roles in pathogen transmission [[77], [78], [79]]. Regions that are geographically closer to one another are generally more likely to experience higher rates of pathogen spread. Additionally, factors such as transportation infrastructure and population density are crucial determinants of transmission dynamics. The “Pathogen + Spatial” model, which accounts for both spatial factors and interpathogen correlations, could provide accurate out-of-sample predictions for malaria transmission [80]. Similarly, a susceptible-exposed-infectious (SEI)–susceptible-exposed-infectious- recovered (SEIR) model that incorporates both seasonal variation in the vector population and short-lived cross-immunity between different DENV subtypes is sufficient to predict epidemics with the characteristic signatures observed in Thailand [81].

### 3.2 Mechanisms of resource competition between coinfection pathogens

Coinfecting pathogens often compete for limited resources within a shared host. When multiple pathogens infect the same individual, they may compete for limited biological resources such as cellular targets, essential nutrients, or replication machinery, leading to antagonistic interactions that suppress one or more pathogens [82].

Spatial competition arises when two or more pathogens attempt to colonize the same anatomical niche. They may compete for epithelial surfaces, cellular niches, or access to immune-privileged sites [[83], [84], [85]]. For example, coinfection of the human lower respiratory tract by IAV and SARS-CoV-2 creates intense competition for AT2 cells, which serve as critical replication sites for both viruses [86].

Nutritional competition may occur when different pathogens rely on the same host-derived substrates. One pathogen may outcompete another by depleting critical resources, as observed in the competition between *Escherichia coli* (*E. coli*) and *Salmonella enterica* (*S. enterica*) in the gut [87]. This competitive advantage is mediated by colicin Ib, a narrow-spectrum toxin produced by *S*. *enterica* that targets closely related *E. coli* strains. Both colicin production and the expression of its receptor CirA are upregulated under inflammation-induced iron limitation, enhancing the ability of *Salmonella* to outcompete *E. coli* in the gut [87].

### 3.3 Modulation of host immunity through interactions

Coinfection with multiple pathogens often alters the host’s immune response, affecting susceptibility to subsequent infections. This may occur through immune suppression, immune evasion, ADE, or even cross-immunity, particularly when pathogens share similar antigens or alleles.

This hypothesis is corroborated by clinical observations, which demonstrate that respiratory viral infections can induce an immune refractory period during which the host is less susceptible to infection by other respiratory viruses [3]. In other words, the prevalence of one respiratory pathogen can block the transmission of another, potentially leading to interference among cocirculating respiratory viruses [88]. The primary mechanism for this viral interference is the interferon response of the host, such as interferon (IFN)-α/β and IFN-λ responses, which enhances antiviral defence and suppresses the replication of other pathogens [88]. This immune response thereby limits the ability of competing viruses to propagate within the host [47].

However, modulation of IFN can paradoxically enhance the transmission [[89], [90], [91], [92]]. For example, *Leishmania* RNA virus 1 or *Phlebovirus* coinfection induces elevated IFN-β expression, which paradoxically facilitates pathogen persistence rather than clearance, likely due to downstream suppression of macrophage activation or altered antigen presentation [93]. Moreover, the upregulation of immune modulators, such as protein kinase R, further contributes to a permissive environment for parasite replication and dissemination [90]. Additionally, coinfection may lead to overactivation of the immune response. Malaria, for example, induces high levels of inflammatory cytokines, such as TNF-α, whereas bacterial infections, such as typhoid fever, can further exacerbate this immune activation. This excessive inflammatory response not only fails to effectively clear pathogens but also causes illness in the host [94].

Cross-immunity is another important aspect of immune modulation and occurs when the immune response against one pathogen provides partial or complete protection against another pathogen. It has been reported that a history of infection with one strain of influenza virus can provide partial immunity against other strains [95]. However, the strength and specificity of cross-immunity can lead to complex epidemiological patterns. In some cases, cross-immunity can drive the evolution of new viral strains that evade existing immunity, as observed in the evolution of influenza viruses and SARS-CoV-2.

The severity of subsequent infections can be increased through ADE. The most established mechanistic explanation for this phenomenon is that differences between pathogens can prevent antibodies from the first infection from neutralizing the second pathogen. Instead, these antibodies may bridge the second pathogen to Fc receptors on immune cells, such as macrophages, facilitating viral entry and leading to ADE of infection and potentially more severe disease [96,97]. In addition to DENV and ZIKV, which we mentioned in Section 2, ADE has been reported in several other pathogens such as Ebola virus [98] and SARS-CoV-2 [99]. However, modelling the effect of ADE remains challenging because of factors such as the structural oversimplification of complex immune interactions, the presence of unquantifiable key parameters, and the difficulty in empirically validating the specific contribution of ADE to disease severity in real-world populations.

## 4 Effect of interactions on disease transmission at the population level

The effects of interactions drive changes in the timing of outbreaks [100] and generate cycles or chaotic oscillations in infection numbers at the population level [101]. Different pathogens may exhibit distinct seasonal or regional transmission patterns, with influenza infection being more prevalent in the winter months and gastrointestinal epidemics occurring predominantly in the summer months [102]. When multiple pathogens coexist, their transmission dynamics can interact, potentially altering the overall transmission patterns. For example, the cooccurrence of certain seasonal infectious diseases may shift their peak transmission periods [103].

The immune response leads to an optimal level of enhancement that prevents pathogen extinction [104], whereas increased mortality may inhibit the coexistence of immunologically similar serotypes [105]. Modelling analysis revealed that cross-immunity dynamics among three respiratory pathogens: RSV, human parainfluenza virus (HPIV), and human metapneumovirus (hMPV), not only affect infection intensity but also alter the seasonal cycles and intervals between outbreaks, with stronger immune protection potentially leading to prolonged infection cycles or reversed dynamics [106]. It has also been shown that the basic reproduction numbers (*R_0_*) of different dengue virus strains are most sensitive to the primary infection rates in humans and mosquito mortality rates [107]. Moderate cross-protective immunity gives rise to persistent out-of-phase oscillations similar to those observed in Bangkok [108].

Additionally, some pathogens may trigger superspreader events in coinfected hosts, facilitating rapid pathogen spread within a short timeframe and increasing the likelihood of outbreaks. Influenza viruses, for example, can cause frequent coughing and sneezing, which facilitate the transmission of other respiratory pathogens, such as bacterial pneumonia. Superspreader events associated with respiratory viruses are often linked to coinfections; thus, when individuals whose nasal passages are colonized by *Staphylococcus aureus* are also infected with mild respiratory viruses, they release large quantities of pathogens in aerosolized bacterial clouds and infect nearby individuals [67].

## 5 Population-level mathematical modelling for interactions: Transmission dynamics and beyond

Mathematical models are indispensable tools for understanding and predicting the dynamics of infectious diseases, particularly when multiple pathogens interact within a population. These models help researchers simulate complex real-world scenarios and assess the effects of various factors on pathogen transmission. The study of pathogen interactions is critical for public health, especially in an era marked by the co-circulation of multiple infectious agents. Different mathematical approaches, such as compartmental, statistical, and network models, offer unique advantages and challenges in exploring these interactions. This section provides an overview of the traditional and contemporary mathematical modelling techniques that are used to investigate multipathogen interactions, highlighting their strengths, limitations, and applications.

The compartmental model divides the host population into different compartments on the basis of their infection status (e.g., susceptible, exposed, infected, or recovered) and establishes connections through cross-infection, coinfection, or other mechanisms to describe pathogen transmission across different populations using different parameters [109]. For example, transmission and susceptibility impacts could be captured by varying transmission rates, whereas pathogenesis impacts could be captured by changes in reporting rates, i.e., if coinfection causes severe disease, cases are more likely to be reported [110]. The compartmental models have been used to examine the stability, periodicity, and critical transmission thresholds of cocirculations, demonstrating how key model parameters influence epidemic spread [81,[106], [107], [108],^111^]. However, the primary limitation of this approach lies in its reliance on overly simplified assumptions, such as homogeneous mixing, which may not fully capture the complexities of real-world disease dynamics. These models typically assume a single transmission route, overlooking the complexity of pathogens that may spread through multiple pathways such as airborne, contact, or vector-borne transmission. Additionally, compartmental models often fail to account for individual-level infection histories or repeated exposures, which can significantly influence susceptibility and immune responses [112,113]. While individual-based models may be better suited for these complex dynamics, they require substantial computational resources and advanced statistical methods for accurate parameter estimation [114,115].

Statistical models utilize time series analysis, regression techniques, Bayesian inference, and other statistical methods to analyze pathogen transmission data. These models provide flexibility in incorporating interaction terms that describe relationships between various factors, such as pathogens, environmental influences, and behavioral changes [113,116]. In statistical models, interaction terms can be represented as product terms, summation terms, or more complex nonlinear functions. For example, the impact of vaccination can be modelled by incorporating terms that simulate its role in controlling epidemic spread [117]. Although statistical models are valuable for identifying correlations between pathogens, they are limited in their ability to infer causal relationships and can suffer from overfitting, especially in the presence of complex conditions and numerous parameters [118]. These models are often built on specific assumptions; for example, regression-based frameworks typically assume linear or stationary relationships between variables, which may not hold true in dynamic disease transmission scenarios [119,120]. To increase the accuracy and reliability of inferences, future applications should integrate interdisciplinary approaches that incorporate insights from epidemiology, data science, and systems biology. Additionally, accounting for external changes (e.g., behavioral shifts, intervention policies, or climate variations) and combining compartmental models with other modelling frameworks, such as agent-based or network models, may provide a more robust understanding of complex epidemic dynamics.

Network models represent pathogen dynamics as a graph, with nodes indicating individuals or pathogens and edges reflecting transmission routes or interactions. This approach is particularly effective in simulating multipathogen interactions, as it allows for flexible representation of the strength and nature of interactions through edge weights and directed relationships. Network models have been used to study epidemic spread dynamics and highlight the critical role of superspreaders in influencing disease transmission [121]. However, network models face several limitations. First, as the network size increases, computational demands increase sharply, posing significant challenges for large-scale simulations. Second, there is an inherent tension between simulating local and global transmission dynamics, as these processes operate through different mechanisms and patterns. Local outbreaks often involve frequent and repeated contacts within closed settings such as communities, schools, or workplaces, leading to clustering effects and rapid spread in confined populations. In contrast, global transmission depends on more complex networks, which are driven by international travel, transnational logistics, and global social connections, with transmission pathways being influenced by mobility patterns, transportation infrastructure, and policy measures, making it difficult to capture both scales accurately within a single model framework. Simplifying the network by removing low-impact nodes and edges can mitigate this challenge, but obtaining reliable parameters for network models remains a significant challenge because of the lack of direct clinical data [122].

The paucity of high-quality epidemiological data further complicates the accurate modelling of interactions. Specifically, gaps remain in our understanding of the prevalence, incidence, and characteristics of pathogens circulating in the community. In particular, detailed surveillance data on different age groups, high-risk populations, and individuals with coinfections involving multiple pathogens are lacking. Additionally, data from surveillance systems are often incomplete, inconsistent, and insufficiently linked at the individual level, which undermines efforts to accurately characterize interactions [123]. Enhanced surveillance systems and improved data quality are crucial for improving model accuracy. Current models lack adequate incorporation of several critical determinants of pathogen dynamics. Specifically, these models often overlook the dynamic interplay between pathogen antigenic evolution and host immune adaptation, which is central to understanding the continuous evolution of pathogens and the unpredictable trajectories of emerging variants. In addition, many models rely on assumptions of stable coexistence and fail to capture the rapid evolution of pathogens in response to shifting immune pressures and environmental factors. Moreover, these models rarely account for the long-term evolutionary effects of public health interventions, such as vaccination campaigns and social distancing measures, on pathogen selection pressures and mutation supply, potentially leading to the underestimation of future infection or mortality risks. Collectively, these limitations reduce the ability of current modelling frameworks to accurately represent the complex and dynamic processes that drive pathogen transmission and evolution.

Despite these challenges, mathematical models remain powerful tools for understanding the dynamics of interactions between pathogens. Our review suggests that these interactions can significantly affect the dynamics of zoonotic diseases, such as SARS-CoV-2 infection, especially when they are combined with other endemic infections. The current models can be extended to incorporate additional complexities, such as age structure, vaccination status, and temporal variations resulting from new variants or changes in control measures.

## 6 Conclusions

Zoonotic infectious diseases remain a critical challenge to global health, with complex interactions between cocirculating pathogens playing significant roles in shaping disease transmission dynamics. The interactions between pathogens—whether synergistic, antagonistic, or neutral—can substantially alter disease outcomes, affecting both the severity of infections and public health responses. As demonstrated by various case studies, these interactions can manifest through multiple mechanisms, including transmission enhancement, immune modulation, and resource competition. Mathematical models provide powerful tools for understanding and predicting the population-level effects of these interactions, enabling more informed disease control strategies.

Future research should explore more integrated, system-level approaches to understand the interactions between multiple pathogens in broader ecological and public health contexts. This could involve not only pathogens that cause respiratory infections but also pathogens that influence other aspects of health. Given the likelihood of cocirculating pathogens in the postpandemic era, these modelling efforts could provide critical insights into optimizing public health strategies and interventions, such as vaccination schedules or public health messaging, in anticipation of future infectious disease threats.
